# KetoCycle mobile app for ketogenic diet: a retrospective study of weight loss and engagement

**DOI:** 10.1186/s40795-022-00539-2

**Published:** 2022-05-02

**Authors:** Sarunas Valinskas, Kasparas Aleknavicius, Justinas Jonusas

**Affiliations:** 1 Faculty of Medicine, Vilnius University, M. K. Čiurlonio Str. 21, 03101 Vilnius, Lithuania; 2 Kilo.Health, Antakalnio g. 17, LT 10312 Vilnius, Lithuania; 3 Lithuania Business University of Applied Sciences, Turgaus st. 21, LT 91249 Klaipeda, Lithuania

**Keywords:** KetoCycle, Mobile application, Weight loss, Obesity, Overweight

## Abstract

**Background:**

The ketogenic diet is one of the oldest diets that has been used for more than a centennial in the clinical setting, and it is gaining popularity as a measure to fight obesity, which is a major predisposing factor for many diseases to manifest, including diabetes mellitus, chronic heart disease, cancer, and others. Thus, we designed this retrospective investigation to determine if users of the mobile application KetoCycle achieved statistically significant weight loss outcomes.

**Methods:**

The initial study cohort comprised 12,965 consecutive users who started using KetoCycle between January 2020 and December 2020. The final cohort comprised 10,269 users. The main parameters obtained from the database containing all self-reported data were gender, number of active days (AD), total time of use (TT), height, initial weight, and last recorded weight. The primary outcome of the study was weight loss. Statistical analyses were performed using IBM SPSS Statistics, version 26 (IBM Corp., Armonk, NY, USA). In addition, a standard multiple regression model was created to predict weight loss from significant actions.

**Results:**

A retrospective analysis of KetoCycle user data showed that 87.3% of KetoCycle users lost some of their initial weight. Of those, 1645 users (18.3%) lost more than 10% of their initial body weight, 3528 (39.3%) users lost between 5 and 10% of their initial body weight, and 3796 (42.3%) users lost less than 5% of their body weight. When user activity was taken into account, it was found that active users lost statistically significantly more weight than non-active users (*p* < 0.05). App engagement was also associated with losing > 5% of initial weight. Using water tracking, weight tracking, and creation of a meals list within KetoCycle statistically significantly predicted weight loss in a multiple regression model.

**Conclusions:**

We concluded that KetoCycle appeared as a promising mobile application suited for weight loss and weight control.

**Trial registration:**

This retrospective chart review study was approved by BRANY IRB in January 2022 (registration ID.: 21-08-564-939).

## Background

The ketogenic diet (KD) is one of the oldest diets that has been used for more than a centennial in the clinical setting [1]. This diet usually consists of a high amount of fats in the rationale, up to 1.5 g of proteins per kilogram of body mass, and is usually highly constrained in the use of carbohydrates, which are allowed up to 50 g per day [2]. Initially, the principles of the KD were utilized by French physicians to control children epilepsy seizures by inducing ketosis by starvation—a metabolic state of the human body when a high amount of ketones are produced from fatty acids during their metabolism in order to provide the energy needed for normal functioning of the human body when the amount of carbohydrates (CHO) is insufficient [3]. During the KD, ketosis is induced by lowering the amount of CHO in the diet to the minimum [4]. Initially, glycemia is maintained by the activation of the gluconeogenesis path and glucose synthesis from glycogen [5]. However, once glycogen reserves are depleted and gluconeogenesis cannot provide enough energy for the body to be fully functional, the primary energy metabolism shifts to the energy production from ketone bodies (KB), which can produce up to two-thirds of ATP that is needed [6, 7]. Moreover, it was shown by Owen et al. that the brain, which is the main glucose user in the human body, can switch to KB such as β-hydroxybutyrate and acetoacetate as the main energy source [8].

Despite the KD’s potential in managing children’s epilepsy, it is gaining popularity as a measure to fight obesity, which is a major predisposing factor for many diseases to manifest, including diabetes mellitus, chronic heart disease, cancer, and others [9]. A systematic article review by Bueno et al. showed that individuals included in KD groups achieved statistically significant body weight loss compared to subjects assigned to low-fat diet groups (weighted mean difference in kilograms: 0.91, 95% CI [0.17–1.65]) [10]. Another systematic review showed similar results—subjects in low-carbohydrate groups lost statistically significantly more weight in comparison to subjects in low-fat diet groups (weighted mean difference in kilograms: 2.17, 95% CI [0.99–3.36]) [11]. Moreover, it has been shown that circulating KB provide even more beneficial effects like protection against cognitive impairment caused by obesity, positive effects on mood, and others [12–14]. On the other hand, it was highlighted that the ketogenic diet also increases the level of low-density cholesterol—an important cardiovascular disease risk factor [11, 15]. Additionally, it has been reported that the KD has some potential drawbacks like headache, fatigue, nausea, constipation, and a temporary decrease in endurance [16]. A recent review by Schutz et al. argues that because of large interindividual variability of metabolic responses to low CHO diets, such diets should be promoted with great care [17].

It is stipulated that one of the significant problems that affect the outcomes and efficacy of ketogenic diet trials is low compliance rates to the prescribed diet [18]. In a meta-analysis by Feng et al., it was reported that compliance rates to the classical ketogenic diet are about 38% [19]. Such low compliance may be related to the restrictiveness of the diet and lack of efficacy [20, 21]. Mobile health (mHealth) applications come into mind as a tool that can help solve this poor compliance problem. It was shown by West et al. that mHealth apps increase users’ motivation and compliance to dietary interventions [22]. One of the apps available in the app stores that targets weight loss while using the KD is KetoCycle. This app provides users with a ketogenic meal plan adapted to their preferences and suggests a home workout and exercise regimen.

Considering that there is a lack of publications related to the weight loss achieved by using the ketogenic diet delivered by a mobile application, we designed a retrospective study to check the efficacy of the mobile application KetoCycle. The primary purpose of this study was to investigate the efficacy of the ketogenic diet delivered through a smartphone application in terms of weight loss and user activity. The secondary purpose of the study was to identify the variables that may impact the weight change.

## Methods

This retrospective chart review study was approved by BRANY IRB in January 2022 (registration ID.: 21-08-564-939). The initial study cohort comprised 12,965 consecutive users who started using KetoCycle between January 2020 and December 2020. After the preliminary screening, users that did not meet all the inclusion criteria were eliminated from further investigation. The inclusion criteria were: 1) the user has entered their gender, height, and initial weight; 2) the user has entered their weight more than once during application usage time; 3) the application was used for more than a month; 4) the user had at least one active day per week during which they documented activities or performed any reasonable app-related actions (e.g., entered anthropometric data, searched for dietary advice, completed exercise from the list, etc.). The final cohort comprised 10,269 users.

The central piece provided by the KetoCycle app is the ketogenic diet meal plan. Nutrition guidelines generally recommend around 2000 cal per day for women and around 2500 for men with daily moderate physical activity levels [23]. When creating individualized meal plans, the focus mainly remains on each person’s anthropometric measurements, such as weight and height, also taking into account gender, age, and physical activity. According to these measurements, the individual calorie needs are calculated. After that, other aspects are considered, such as a person’s food preferences, health goals, and underlying conditions or diseases they might have. Most calories come from fat (~ 60%), a moderate amount from proteins (~ 30%), and the rest from carbohydrates. Unsaturated fats are prioritized over saturated fats, keeping the latter to < 10% of caloric contribution. The users are able to choose which products that are compatible with the KD they would like to include in their meal plan according to their preferences. Furthermore, KetoCycle provides users with daily educational material. The app can also remind users to input their meals and regularly track their weight, daily water intake, and steps, and gives the possibility to set personal goals and review their progress. Finally, a workout plan can be created for each user according to their self-determined fitness level, with explanations and video demonstrations of each exercise. The workouts are based on high-intensity interval training. The rxercises in the workouts are selected according to a person’s fitness level and medical condition.

The main parameters obtained from the database containing all self-reported data were gender, number of active days (AD), total time of use (TT), height, initial weight, and last recorded weight. For this study, we assumed that active days (AD), through more responses to app reminders and more regular in-app activities, represented better adherence to the provided diet plan. In addition, significant actions, such as water and weight tracking, completed workout count, and number of created shopping and meal lists, were also recorded. If the number of performed operations for a specific activity was higher than the cohort’s median for that activity, the user was considered active in that regard. Dummy variables for different activities were created later. If the user was active in a specific activity, the new dummy variable was coded as “1”, while inactivity was coded as “0”. If the subject’s AD was higher than the top 25th percentile, they were considered an active user.

Starting and final body mass indexes (BMI) were calculated from the reported data. BMI levels were determined according to CDC standards: > 25 and ≤ 30 kg/m^2^: overweight; > 30 and ≤ 35 kg/m^2^: Obesity Class 1; > 35 and ≤ 40 kg/m^2^: Obesity Class 2; and > 40 kg/m^2^: Obesity Class 3 [24]. The users were allocated into groups based on their percentage of lost weight (Group 1: > 10%; Group 2: between 5 and 10%; Group 3: less than 5%; and Group 4: weight increased).

Statistical analyses were performed using IBM SPSS Statistics, version 26 (IBM Corp., Armonk, NY, USA). As a first step, the normality of the data was checked by inspecting box and Q-Q plots, calculating the Z-value of kurtosis and skewness, and using the Shapiro–Wilk test. Data with a normal distribution are presented as means with standard deviations (SD), and data with a non-normal distribution as medians with ranges. Categorical data are shown as a number with the percentage in parentheses. The difference between means of starting and last recorded data with a normal distribution was compared using t-test statistics. If the data had a non-normal distribution, the means between baseline and final data in related samples were compared using the Wilcoxon signed-rank test, while means between independent samples were compared using the Mann–Whitney U test. Additionally, Spearman’s r correlation was used to determine the relationship between weight loss, AD, and TT. Finally, the chi-square test was used to find associations between body weight loss and engagement groups. The significance level was chosen to be 0.05 in all tests.

Finally, a standard multiple regression model was created to predict weight loss from significant actions (weight, water tracking, etc.). Linearity was assessed by partial regression plots and a plot of studentized residuals against the predicted values. The independence of residuals was assessed using the Durbin–Watson statistic.

## Results

The study cohort predominantly comprised females (81.7%). The majority of users that started using KetoCycle had overweight (35.1%) or obesity (Class I, 31.8%). On the other hand, 12.1% of the users had normal weight at the start of app use. There was a statistically significant initial weight and BMI difference between male and female cohorts. There were no statistically significant differences between medians of active days and the total time of use in female and male cohorts (*p* > 0.05). Detailed descriptive statistics are shown in Table 1.

**Table 1:** Detailed descriptive statistics of KetoCycle user data at baseline. Means are shown with standard deviations (SD), while medians are shown with ranges in parentheses. *p*-values < 0.05 are bolded

Number of users	10,269	Significance
Gender, No. (%)		
Male	1875 (18.3%)	*p* **<** **0.001**
Female	8394 (81.7%)	
User distribution among different initial weight groups, No. (%)		
Normal weight	1246 (12.1%)	*p* > 0.05
Overweight	3601 (35.1%)	
Obese, Class I	3261 (31.8%)	
Obese, Class II	1597 (15.6%)	
Obese, Class III	564 (5.5%)	
Height, cm ± SD		
Overall	166.18 ± 8.39	***p*** **< 0.001**
Male	178.68 ± 6.92	
Female	163.83 ± 6.74	
Weight, kg ± SD		
Overall	85.39 ± 16.77	***p*** **< 0.001**
Male	101.59 ± 15.19	
Female	81.78 ± 14.86	
BMI ± SD		
Overall	30.83 ± 5.08	***p*** **< 0.001**
Male	32.52 ± 4.45	
Female	30.45 ± 5.14	
Active days of use, days (range)		
Overall	45 (30–100)	*p* > 0.05
Male	45 (30–100)	
Female	45 (30–100)	
Total time of use, days (range)		
Overall	184 (30–574)	*p* > 0.05
Male	184 (30–566)	
Female	184 (30–574)	

The body weight change was inspected to investigate the users’ weight loss while using KetoCycle. It was found that 87.3% of the users (*N* = 8969) lost a part of their initial weight during the study time. On the other hand, 745 users (7.3%) retained the same weight, while 555 (5.4%) users increased their initial body weight. Of those who managed to reduce their weight, 1645 users (18.3%) lost more than 10% of their initial body weight, 3528 (39.3%) users lost between 5 and 10% of their initial body weight, and 3796 (42.3%) users lost less than 5% of their body weight. When bodyweight change was analyzed in general and in male and female cohorts separately, it was found that there was a statistically significant body weight loss in the general cohort (from 85.39 ± 16.77 kg to 80.41 ± 15.57 kg; *p* < 0.05). Statistically significant body weight loss was also observed in the male (from 101.59 ± 15.19 to 94.67 ± 14.17; *p* < 0.05) and female (from 81.78 ± 14.86 to 77.22 ± 14.00; *p* < 0.05) cohorts. It is worth mentioning that more than one-third of the users reduced their BMI level (*N* = 3636), but only those with overweight or obesity. No users reduced their body mass index below 18.5 kg/m^2^ during app use.

Usage trend analysis followed. Associations between weight change and the total time of use and weight change and active time were checked. It was found that there was a statistically significant moderate positive correlation between active time and weight change (rs = 0.177, *p* < 0.05). Interestingly, there was a very weak negative association between total time of use and weight change, but it was statistically insignificant (rs = − 0.037, *p* > 0.05). Therefore, only the measure of active time was used in further statistical analyses. A moderate positive correlation was found between weight loss and activity score (rs = 0.192, *p* < 0.05).

Subjects were assigned to engagement groups following the methodology described previously. Bodyweight loss among the different engagement and starting BMI level groups was analyzed. Statistically significant body weight loss was found in all engagement groups among five different initial BMI level cohorts (Table 2). Moreover, we found that starting bodyweights between the users in the different engagement groups did not differ statistically significantly. At the same time, there was a statistically significant difference in final body weight in all BMI level cohorts except in a group of users with severe obesity (Obesity Class III). However, there was a statistically significant trend that people in the active group lost more weight during app use. Moreover, there was a strong positive association between starting BMI level and weight loss (rs = 0.314, *p* < 0.05)—users with higher initial weight tended to lose more weight while using the app.

**Table 2:** Body weight loss among different starting BMI levels and engagement groups. Means are shown in kilograms ± SD

Users with normal weight						
Engagement group	Initial weight		Final weight		Mean diff.	Significance
Non-active	64.26 ± 6.69	*p* = 0.177	61.85 ± 6.54	*p* = 0.010	2.41 ± 0.08	*p* = 0.000
Active	63.65 ± 6.63		60.69 ± 7.03		2.96 ± 0.19	*p* = 0.000
					0.55 ± 0.21	*p* = 0.010
Users with overweight						
Engagement group	Initial weight		Final weight		Mean diff.	Significance
Non-active	76.45 ± 8.83	*p* = 0.141	72.79 ± 8.99	*p* = 0.000	3.65 ± 0.07	*p* = 0.000
Active	75.94 ± 8.84		70.84 ± 9.06		5.10 ± 0.15	*p* = 0.000
					1.45 ± 0.16	*p* = 0.000
Users with Obesity, Class I						
Engagement group	Initial weight		Final weight		Mean diff.	Significance
Non-active	89.66 ± 10.31	*p* = 0.893	84.21 ± 10.16	*p* = 0.001	5.04 ± 0.09	*p* = 0.000
Active	89.72 ± 10.28		83.27 ± 10.56		6.45 ± 0.19	*p* = 0.000
					1.40 ± 0.21	*p* = 0.000
Users with Obesity, Class II						
Engagement group	Initial weight		Final weight		Mean diff.	Significance
Non-active	103.47 ± 11.86	*p* = 0.705	96.97 ± 11.81	*p* = 0.001	6.49 ± 0.16	*p* = 0.000
Active	103.22 ± 11.56		94.77 ± 11.94		8.45 ± 0.29	*p* = 0.000
					1.95 ± 0.33	*p* = 0.000
Users with Obesity, Class III						
Engagement group	Initial weight		Final weight		Mean diff.	Significance
Non-active	114.16 ± 11.11	*p* = 0.146	106.49 ± 11.11	*p* = 0.140	7.67 ± 0.29	*p* = 0.000
Active	115.64 ± 10.20		104.97 ± 10.81		10.67 ± 0.51	*p* = 0.000
					3.00 ± 0.57	*p* = 0.000

Afterward, the distribution of active and non-active users among the weight loss groups was observed by creating a crosstabulation between the groups (Table 3). There was a statistically significant association between body weight loss and usage groups (χ2 = 280.78, *p* = 0.000, Cramer’s V = 0.165). The adjusted residuals showed an increase in the count of users who lost more than 5% of their initial body weight. Meanwhile, the number of users who lost less than 5% or increased their initial body weight was increased in the non-active group.

**Table 3:** Crosstabulation of body weight loss and engagement groups. Adjusted residuals appear in the parentheses next to the observed frequencies

Bodyweight loss groups				
Usage group	Lost > 10%	Lost > 5% but less than 10%	Lost < 5%	Increased
Non-active	990 (−14.8)	2581 (−2.7)	3073 (11.1)	1030 (4.0)
Active	655 (14.8)	947 (2.7)	723 (−11.1)	270 (−4.0)

Finally, a linear regression model was created to determine which activities may have contributed the most to weight loss (Table 4). The multiple regression model statistically significantly predicted weight loss of subjects who were using KetoCycle (F(5,10,263) = 201.059, *p* = 0.000). Variables that statistically significantly predicted weight loss were water tracking, weight tracking, and creation of a meals list. Completed workouts did not contribute to the model significantly. However, it is worth mentioning that creating a shopping list statistically significantly contributed to the increase of weight. Meanwhile, weight tracking was the most contributing event to weight loss. Regression coefficients and standard errors can be found in Table 4.

**Table 4:** Multiple regression results for weight loss

	B	95% CI for B		SE	Significance
Model					
Constant	4.047	3.912	4.182	0.069	0.000
Water tracking	0.302	0.088	0.516	0.109	0.006
Weight tracking	3.091	2.881	3.302	0.107	0.000
Workout completion	0.048	−0.253	0.349	0.153	0.755
Creation of a shopping list	−0.280	− 0.486	− 0.074	0.105	0.008
Creation of a suggested meals list	0.480	0.272	0.688	0.106	0.000

## Discussion

The principal finding of our investigation is that the majority of KetoCycle users lost more than 5% of their initial body weight. This is notable because according to CDC recommendations for weight loss, even a 5–10% weight reduction for those with overweight is likely to give health benefits such as improved blood pressure and cholesterol profile [25].

Multiple studies have shown that ketogenic diets can lead to significant weight reduction [26]. A meta-analysis of randomized controlled trials using a ketogenic diet has demonstrated that a ketogenic intervention usually leads to significant weight loss (weighted mean difference of very low carbohydrate ketogenic diet: 0.91, 95% CI [0.17–1.65] kg) [10]. This weight reduction has also been replicated in a ketogenic diet trial using a mobile app [27]. A group that had a ketogenic diet intervention lost 5.6 kg of their initial body weight after 12 weeks (95% CI [4.5–6.7] kg). Moreover, the ketogenic diet group loss statistically significantly more weight than the low-fat diet group did during the intervention time (difference: 3.1 kg; 95% CI [1.5–4.6] kg; *p* < 0.001). We do agree with previous studies showing that KD can help lose a statistically significant amount of weight. In the current study we have found that users in all BMI and engagement groups, including normal-weight users and non-active users, lost statistically significant amounts of weight. Moreover, the general trend was that the higher the initial weight, the more weight was lost. We stipulate that this weight loss came from the changes in resting energy expenditure. It was shown by Ebbeling et al. that resting energy expenditure is greater in a very-low carbohydrate group when compared to that in a low-fat diet group [28].

King et al. report that smartphones can act as platforms that effectively deliver health interventions [29]. West et al. argue that by improving goal-setting and motivation and providing relevant educational material, mobile apps can facilitate diet-related behavior change [22]. Furthermore, a pilot randomized controlled trial has shown that diet adherence and results are better when facilitated by mobile applications [30]. In a systematic review by Liu et al., the authors concluded that certain mobile app-related intervention features, such as automated feedback, reminders, goal-setting, educational materials, and data visualization, support self-management of chronic conditions related to overweight and obesity [31]. KetoCycle also provides features that may be beneficial to weight loss. Alongside the meal plans and educational material regarding a ketogenic diet, the app gives regular automated reminders, provides diet and exercise goal setting and relevant feedback, and visualizes the user’s achievements. As discussed above, such functionalities may help improve KetoCycle users‘motivation and help them adhere to their plan. Additionally, KetoCycle features such as goal setting (i.e., challenges) and corresponding feedback are aspects of “gamification”, which is a rapidly growing trend in mHealth app design. Miller et al. point out that integrating such features brings a certain appeal that improves usability and promotes self-management [32].

Previous studies have found that engagement is an important contributor to weight loss with mHealth applications [33, 34]. A large study that also investigated factors that contribute to weight loss when using an mHealth application reported that in-app exercise logging is a significant predictor [35]. We have also found a similar trend: users who were defined as active lost statistically significantly more weight than those who were non-active in all BMI groups. Moreover, active users were more likely to achieve clinically significant (≥5%) weight loss. We explored this further using a multiple regression model and found that most in-app activities predicted weight loss with varying contributions. Interestingly, workouts logged in the app did not predict weight loss, even though physical activity is a central piece in most weight loss interventions [36]. As there is little doubt that exercise promotes weight loss, the workouts mechanism in KetoCycle warrants further investigation in future studies. However, there are relatively strong data indicating that ketogenic diets reduce weight mainly through body fat loss, sparing lean mass [37]. A possible explanation could be that users who were most engaged with in-app exercises lost mostly body fat while preserving, or even gaining, more muscle mass. Another interesting finding is that creation of a shopping list in the app statistically significantly predicted not weight loss, but weight gain. It is worth noting here that this engagement variable is uncommon in similar mHealth app studies, so its validity is uncertain. It may be worthwhile to investigate its effectiveness and interaction with other variables in upcoming studies. Research regarding which app features are most effective for behaviour change, and how to optimize and deliver them best, is novel and relatively scarce. A common framework for defining and measuring user engagement in wellness apps would also be beneficial. Future studies in mHealth for weight loss should consider including engagement analyses as one of their objectives.

The strengths of this study are the relatively large user sample and analysis of user engagement and app feature contribution. The retrospective nature of our study allowed us to access a relatively large data pool, which increased the strength of our results. Stratifying users into engagement levels and analyzing the predictive effect of app functionalities adds to knowledge regarding mHealth app feature development. The current study also has several limitations. There was no control group and randomization, and the data were self-reported, which currently limits our ability to draw strong conclusions that the observed results were solely due to the use of KetoCycle. There are multiple covariates that may influence the results of weight loss, such as dietary and exercise habits and certain comorbidities. We also did not know the users’ body composition and thus could not conclude whether the observed weight reductions mainly occurred through loss of body fat or lean body mass. Moreover, we analyzed only the first and last weight entries, taking raw weight change into account and ignoring app usage time. Further study should take into account the usage time and look for a difference between the short- and long-term users and how their weight changes during the usage time. It is worth mentioning that as this study was retrospective, we were not actively monitoring users. By that, we might have been missing important data about their activities beyond app use which might lead to significant weight loss or weight gain. Furthermore, we used proxies for action tracking—the fact that the user marked in the app that they completed an activity or consumed a specific food did not mean that they really did it, which may have led to discrepancies in the obtained data.

Additionally, despite the promising initial results, a longer study period is required to assess the sustainability of the observed weight loss. It was noted in a systematic review that relatively large weight loss results facilitated through mHealth interventions tend to attenuate in the longer term [38]. Furthermore, although we did have success with the chosen engagement variables in our regression model, definitions of “activeness” in mHealth apps, including that in this study, are varied and require more validation and a comprehensive underlying framework. Moreover, our study may have been affected by the healthy responder bias [39]. A longer, prospective controlled study with KetoCycle is planned in the near future to expand upon the results herein and address the abovementioned limitations.

## Conclusions

A retrospective analysis of KetoCycle user data showed that users lost a statistically significant amount of body weight. The majority of users lost at least 5 % of their initial body weight. A notable percentage of users lost 10 % or more. It was noted that users with higher initial BMI lost more weight. When user activity was taken into account, it was found that active users lost statistically significantly more weight than non-active users did. App engagement was also associated with losing at least 5 % of initial weight. Using water tracking, weight tracking, and creating a meals list within KetoCycle statistically significantly predicted weight loss in a multiple regression model. However, the current study did not address the exact mechanism and covariates that influenced the weight loss of application users. A prospective study is planned to expand upon the results herein and address the abovementioned limitations.

## Data Availability

The datasets generated and analyzed during the current study are not publicly available due to protection of confidential information of Kilo Health business and Kilo Health users, but are available from the corresponding author upon reasonable request.

## Abbreviations

KD: Ketogenic diet
CHO: Carbohydrates
KB: Ketone bodies
AD: Number of active days
TT: Total time of use
BMI: Body mass index
SD: Standard deviation
